# *Anisakis* allergy versus gastric anisakiasis: A case of repeated *Anisakis*-associated symptoms

**DOI:** 10.1016/j.jacig.2024.100207

**Published:** 2024-01-06

**Authors:** Yuto Hamada, Satoshi Sugano, Yosuke Kamide, Kiyoshi Sekiya, Yuma Fukutomi

**Affiliations:** a Clinical Research Center for Allergy and Rheumatology, National Hospital Organization Sagamihara National Hospital, Sagamihara, Japan; b Department of Gastroenterology, National Hospital Organization Sagamihara National Hospital, Sagamihara, Japan

**Keywords:** *Anisakis* allergy, *Anisakis*, gastric anisakiasis, anisakiasis, anaphylaxis

## Abstract

A 53-year-old patient experienced 2 *Anisakis*-induced allergic episodes: the first with anaphylaxis, the second presenting with gastric symptoms and progressing to systemic anaphylaxis. The case could suggest a common pathophysiology involving allergic reactions in gastric anisakiasis and *Anisakis* allergy.

*Anisakis* is a nematode parasitizing most sea fish and cephalopods worldwide.^1^^,^^2^ Live *Anisakis* larvae also cause parasitic infections in humans, with gastric anisakiasis as one of the common *Anisakis* infections.^2^^,^^3^ The standard treatment is endoscopic removal of the larvae.^2^^,^^3^ Additionally, *Anisakis* causes IgE-mediated allergic reactions, including systemic anaphylaxis subsequent to oral exposure to allergenic proteins derived from *Anisakis* larvae that contaminate seafood in sensitized individuals, as well as occupational allergies in fish processing workers.^4^ In particular, live *Anisakis* larvae have been known to induce IgE-mediated allergic reactions and digestive symptoms.^5^ The treatments for this type of *Anisakis* allergy include intramuscular adrenaline, antihistamines, and steroids that differ from those for anisakiasis, which is an infectious disease.^5^

However, gastric anisakiasis and *Anisakis* allergy share some similarities. The immune response of T_H_2 cells and high levels of specific IgE to *Anisakis* has been documented.^1^^,^^2^ Therefore, systemic corticosteroids, which are antiallergic drugs, reportedly improve gastric anisakiasis.^6^ These diseases also show some similarity in terms of a clinical symptom. Asaishi et al reported that 8.4% of their patients with gastric anisakiasis had urticaria.^7^ Meanwhile, abdominal symptoms are common in patients with *Anisakis* allergy who are experiencing *Anisakis*-induced systemic anaphylaxis.^5^^,^^8^ In the late 1990s, Spanish investigators proposed the concept of gastroallergic anisakiasis (GAA), which is a more specific disease entity for *Anisakis* allergy and a borderline concept between parasitic disease and food allergy.^9^ In patients with GAA, an acute systemic allergic reaction occurs only when live *Anisakis* larvae infest them after ingestion of raw seafood.^5^^,^^9^ In this condition, exposure and sensitization to *Anisakis* can occur when live *Anisakis* larvae penetrate the human mucosa. Therefore, to prevent symptom recurrences, patients with GAA should avoid seafood parasitized by live *Anisakis* larvae.^5^^,^^9^^,^^10^

However, the naming of these anisakiasis-related disease entities and defining of their underlying pathogenesis is still controversial. The insight that they share a common pathophysiology but incidentally have different clinical manifestations remains unconfirmed. Perhaps, patients with gastric anisakiasis develop anaphylaxis after *Anisakis* reinfection and the pathophysiology of abdominal symptoms in gastric anisakiasis is identical to that induced by *Anisakis*-related anaphylaxis in *Anisakis* allergy. We have recently experienced a case that is very thought provoking from the standpoint of understanding the pathogenesis of anisakiasis-associated allergic disease. In this case, repeated *Anisakis*-associated symptoms occurred with an endoscopically proven *Anisakis* infection.

A 53-year-old Japanese female patient with a history of systemic urticaria after ingesting sashimi (raw fish slices) was referred to our department. Her level of *Anisakis*-specific IgE (as determined by ImmunoCAP [Thermo Fisher Scientific, Waltham, Mass]) was 246 kUA/L. After other food and seafood allergies had been ruled out, the patient was diagnosed with *Anisakis* allergy and received an epinephrine autoinjector device for the treatment of anaphylaxis. Unfortunately, she did not adhere to the dietary restriction of avoiding raw seafood intake. At age 55 years, she complained of epigastric pain, nausea, systemic urticaria, and dyspnea with stridor 2 hours after consuming raw squid slices and presented to our department without using an epinephrine autoinjector device (first episode [Fig 1, *A*]). Thus, she was diagnosed with anaphylaxis and treated with intramuscular adrenaline (0.3 mg), which improved her symptoms immediately. Gastroendoscopy was performed 3 hours after the adrenaline treatment; it revealed an *Anisakis* larva invading the gastric cardia. Subsequently, the penetrating larva was successfully removed with biopsy forceps through an endoscopic procedure (see Fig E1, *A* in the Online Repository at www.jaci-global.org). In addition, skin prick testing was performed using the crude extract of the *Anisakis* nematode because allergen extracts from *Anisakis* larvae are not commercially available in Japan. The test result was positive (see Fig E1, *B*). The patient’s level of *Anisakis*-specific IgE was 26.0 kUA/L at the time of anaphylaxis, subsequently increasing to 274 kUA/L at the 1-month follow-up visit.

**Fig 1:**
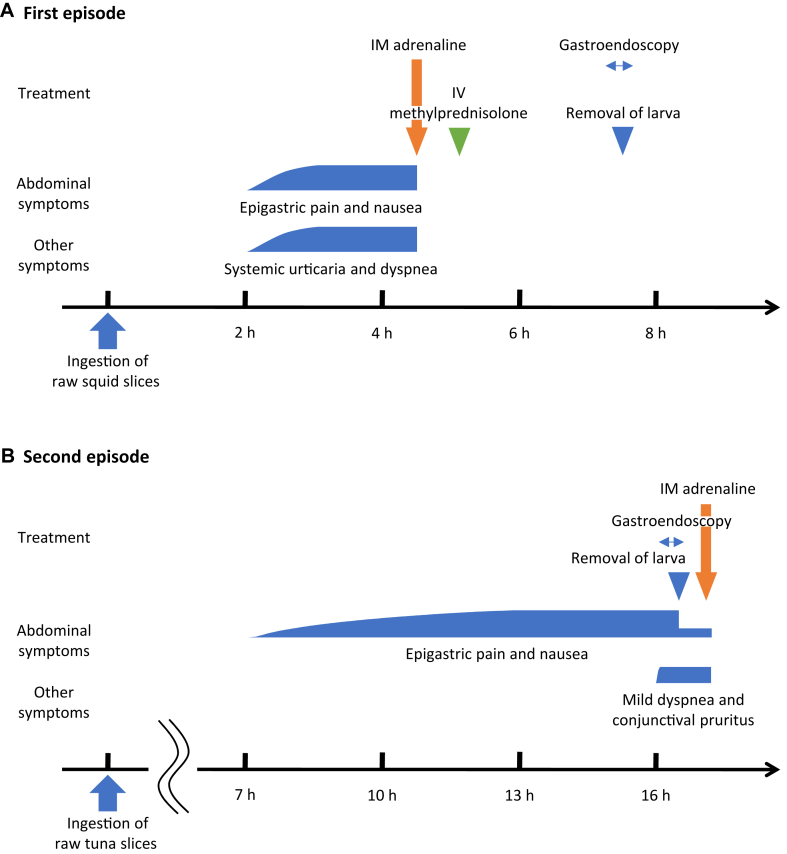
Clinical course of the first episode in the patient at age 55 years (**A**), and the second episode at age 57 years (**B**). Each course of *Anisakis*-associated symptoms is shown with the response to the intramuscular adrenaline and endoscopic removal of an *Anisakis* larva. *IM*, Intramuscular; *IV*, intravenous drip infusion.

At age 57 years, the patient once again complained of epigastric pain and nausea—this time 7 hours after consuming raw tuna slices (second episode [Fig 1, *B*]). Physical examination revealed epigastric tenderness. The gastric antrum in the abdominal computed tomography scan exhibited edematous wall thickening (see Fig E1, *C*). Hence, gastric anisakiasis was suspected. and an emergent gastroendoscopy was performed. Consequently, an *Anisakis* larva invading the gastric antrum was detected and successfully removed endoscopically (see Fig E1, *D*). However, we noted wheezing and conjunctival congestion immediately after the endoscopy procedure. Just before the start of the endoscopy procedure (∼9.5 hours after the gastric symptom onset), the patient reported beginning to feel mild dyspnea and conjunctival pruritus. Thus, we injected adrenaline (0.3 mg) intramuscularly. After 10 minutes, all of her symptoms, including her gastric symptoms, disappeared immediately and completely. Her level of *Anisakis*-specific IgE at the time of anaphylaxis was 28.6 kUA/L, and it subsequently increased to 48.1 kUA/L at the 1-month follow-up visit.

The clinical course of this case is very informative in understanding the pathophysiology of *Anisakis*-associated diseases. This case report is valuable because *Anisakis* infections were endoscopically proved for both episodes experienced by the patient. The first episode was a typical systemic anaphylaxis that was completely improved by intramuscular adrenaline injection, and the cause could have been *Anisakis* infection. This episode is in line with the disease concept of GAA.^5^^,^^9^

Conversely, in the patient’s second episode, she initially complained of gastrointestinal symptoms only, suggesting gastric anisakiasis. However, systemic allergic symptoms ultimately appeared 9.5 hours after the start of abdominal symptoms (16.5 hours after the patient had consumed raw tuna slices), but all of these symptoms were immediately treated with intramuscular adrenaline. Thus, the series of symptoms (abdominal symptoms + systemic allergic symptoms) might have been induced by an IgE-mediated allergic reaction to *Anisakis* infested on the gastric mucosa. Had the *Anisakis* larva possibly been spontaneously expelled, the patient may not have experienced systemic allergic symptoms. Additionally, her abdominal symptoms indicating gastric anisakiasis might have been improved had we injected intramuscular adrenaline even before systemic allergic symptoms occurred and even before the larva was removed endoscopically, as evidenced by the observed improvement in the abdominal symptoms after adrenaline treatment before the endoscopic removal of *Anisakis* larvae in the first episode.

In conclusion, gastric anisakiasis and *Anisakis* allergy may possibly occur as a syndrome with different clinical manifestations induced by a common pathophysiology—that is, an immediate allergic reaction induced by *Anisakis* infection on the gastric mucosa. Clinical feature studies using a large number of cases are needed for further examination.

## Disclosure Statement

This study was partly supported by research funding from the JSA WAO 2020 Memorial Research Grant Program by the Japanese Society of Allergology.

Disclosure of potential conflict of interest: The authors declare that they have no relevant conflicts of interest.
