# Supplementary material for: Anisakis allergy versus gastric anisakiasis: A case of repeated Anisakis-associated symptoms
Source: J Allergy Clin Immunol Glob. 2024 Jan 6;3(2):100207. doi: 10.1016/j.jacig.2024.100207 (PMC10838902; doi:10.1016/j.jacig.2024.100207)
Supplement: Supplementary Figure [file mmc1.pptx]

## Slide 1
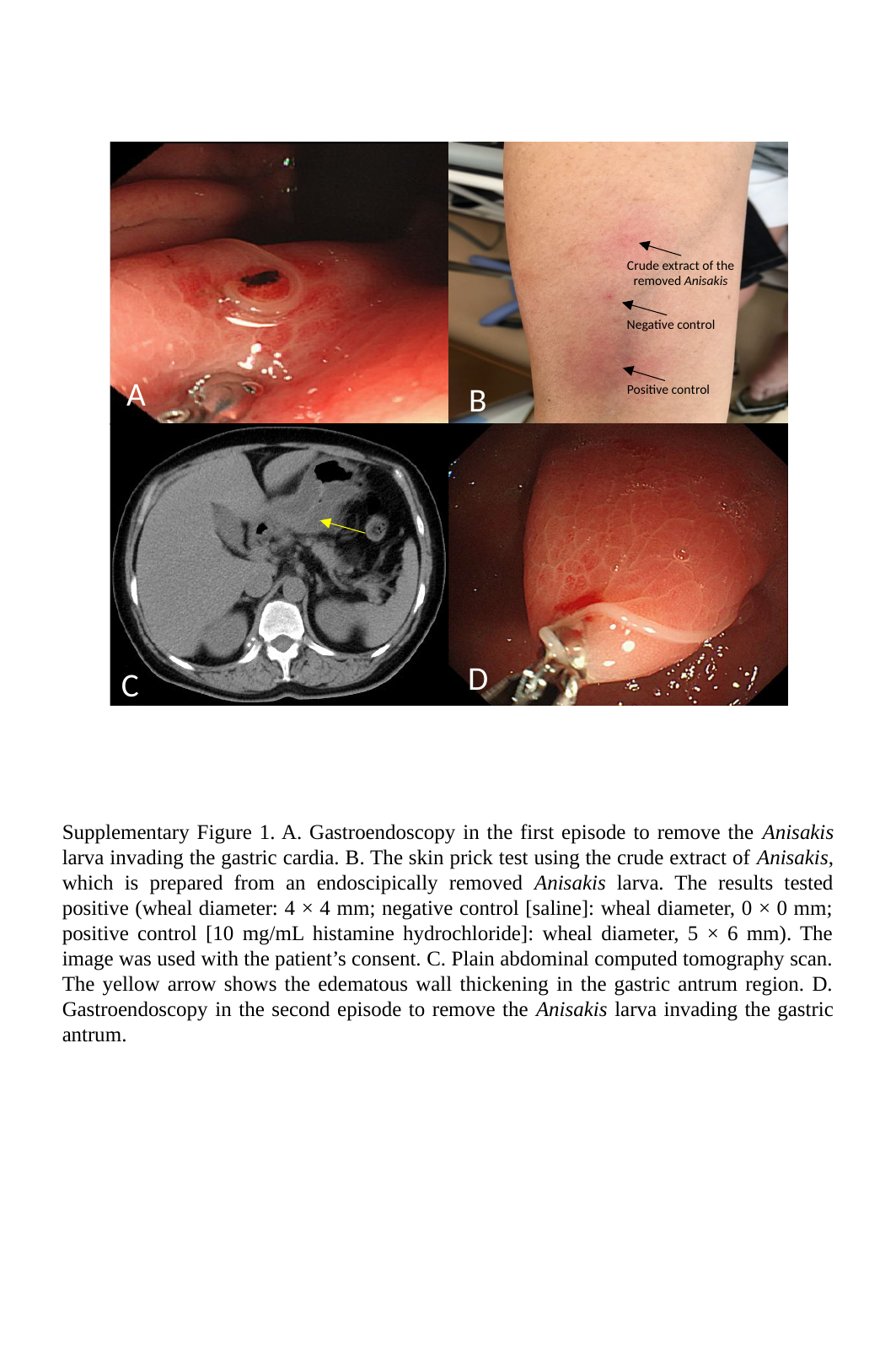

Crude extract of the removed Anisakis
Negative control
A
Positive control
B
D
C
Supplementary Figure 1. A. Gastroendoscopy in the first episode to remove the Anisakis larva invading the gastric cardia. B. The skin prick test using the crude extract of Anisakis, which is prepared from an endoscipically removed Anisakis larva. The results tested positive (wheal diameter: 4 × 4 mm; negative control [saline]: wheal diameter, 0 × 0 mm; positive control [10 mg/mL histamine hydrochloride]: wheal diameter, 5 × 6 mm). The image was used with the patient’s consent. C. Plain abdominal computed tomography scan. The yellow arrow shows the edematous wall thickening in the gastric antrum region. D. Gastroendoscopy in the second episode to remove the Anisakis larva invading the gastric antrum.
